# Bacterial Infections in Decompensated Cirrhosis: Clinical Profile, Microbiology, and Antimicrobial Resistance Patterns

**DOI:** 10.7759/cureus.108810

**Published:** 2026-05-13

**Authors:** Anubhav Dabas, Mohmad Sejarali Sayeed, Praful Kumar Chavhan, Tehsin Saiyad, Jaskirat K Hundal, Tazean Zahoor Malik, Sajidali S Saiyad, Gajendra Anuragi, Avinash Kumar Sudan

**Affiliations:** 1 Gastroenterology, Oxygen Hospital, Rohtak, IND; 2 Gastrointestinal Surgery, Aryavart Hospital, Meerut, IND; 3 Gastroenterology, Aryavart Hospital, Meerut, IND; 4 Microbiology, Veer Narmad South Gujarat University, Surat, IND; 5 Clinical Sciences, Washington University of Health and Science, San Pedro, BLZ; 6 Community Medicine, Sher-i-Kashmir Institute of Medical Sciences (SKIMS), Srinagar, IND; 7 Physiology, Pacific Medical College and Hospital, Pacific Medical University, Udaipur, IND; 8 Surgical Gastroenterology, Madras Medical College, Chennai, IND; 9 Gastroenterology, Maharishi Markandeshwar Institute of Medical Sciences and Research, Mullana, IND

**Keywords:** antibiotic stewardship, antimicrobial resistance, bacterial infections, clinical outcomes, decompensated cirrhosis, india, multidrug-resistant organisms, spontaneous bacterial peritonitis

## Abstract

Background

Hospitalised patients with decompensated cirrhosis face a high risk of bacterial infections. Antimicrobial resistance (AMR) is increasing globally, yet prospective data from the Indian subcontinent - a recognised AMR hotspot - remain limited.

Methods

This prospective observational study enrolled 176 consecutively hospitalised adults with decompensated cirrhosis over 18 months (September 2022 to February 2024). We analysed clinical profiles, infection prevalence, microbiological isolates, antimicrobial susceptibility patterns, and in-hospital outcomes. Multivariate logistic regression identified independent predictors of infection and mortality.

Results

Bacterial infections were documented in 82 patients (46.6%). Infected patients were younger (mean age, 48.5 vs. 53.8 years) and predominantly male (76.8%), with higher disease severity (93.9% Child-Turcotte-Pugh (CTP) class C; 79.3% Model for End-Stage Liver Disease-sodium (MELD-Na) >21). In-hospital mortality was significantly higher in infected patients (31/82, 37.8%) compared with non-infected patients (3/94, 3.2%; p < 0.001). Culture positivity was 48.8% (40/82), with *Escherichia coli* (27.5%) and *Klebsiella* spp. (17.5%) predominating. Overall, drug resistance was 57.5%, comprising 45.0% multidrug-resistant (MDR) and 12.5% extensively drug-resistant (XDR) organisms. MDR prevalence was highest in urinary tract infections (UTIs) (72.7%), while XDR was most frequent in spontaneous bacterial peritonitis (SBP) (33.3%). Susceptibility of *E. coli* to ceftriaxone was only 27.3%. Elevated international normalised ratio (INR) and hypoalbuminemia independently predicted infection, whereas hyperbilirubinemia predicted mortality.

Conclusion

Bacterial infections affect nearly half of hospitalised patients with decompensated cirrhosis in North India and are associated with markedly poor outcomes. The alarmingly high prevalence of MDR and XDR organisms renders conventional empirical antibiotics inadequate. Region-specific antibiograms, enhanced antimicrobial stewardship, and infection prevention strategies are urgently needed in this vulnerable population.

## Introduction

Cirrhosis of the liver represents the end-stage consequence of chronic liver injury, characterised histologically by regenerative nodules surrounded by fibrous bands that lead to portal hypertension and progressive hepatic dysfunction [1]. The clinical course follows a biphasic trajectory: an initial compensated phase, often asymptomatic, with a median survival of 10-12 years, followed by a decompensated phase marked by overt clinical complications such as ascites, variceal bleeding, encephalopathy, and jaundice [2].

Bacterial infections constitute one of the major clinical challenges in patients with decompensated cirrhosis [3]. Infection prevalence in hospitalised cirrhotic patients ranges from 25% to 47%, representing a four- to five-fold higher risk compared with non-cirrhotic hospitalised patients [4]. This susceptibility stems from multiple mechanisms: liver dysfunction, portosystemic shunting, gut dysbiosis, increased bacterial translocation, cirrhosis-associated immune dysfunction (CAID), and genetic predisposition [5]. The immunocompromised state in advanced liver disease involves both innate and adaptive immune systems, creating a permissive environment for microbial invasion [6].

Bacterial infections in cirrhotic patients often present subtly, requiring a high index of suspicion. The Baveno VII guidelines recommend that all hospitalised patients with decompensated cirrhosis be considered potentially infected and undergo appropriate diagnostic evaluation [7]. However, cultures are positive in only 50%-70% of cases, limiting diagnostic certainty and complicating management [8].

The microbiological landscape has undergone significant epidemiological shifts. Current data show re-emergence of gram-negative bacteria alongside rising multidrug-resistant (MDR) organisms [3,9]. *Escherichia coli* remains the most frequent gram-negative isolate, while *Enterococcus* spp. and *Staphylococcus aureus* predominate among gram-positive isolates [10].

Antimicrobial resistance (AMR) is an escalating concern in cirrhosis, with MDR organisms increasingly prevalent, particularly in the Indian subcontinent [11]. The EPIC-AD study from North India reported MDR organisms in 71% of culture-positive infections, including 48% carbapenem-resistant isolates [12]. Rising resistance to standard empirical agents, such as third-generation cephalosporins and fluoroquinolones, is increasingly compromising treatment strategies [13].

Bacterial infections in cirrhosis have severe clinical consequences, triggering inflammatory responses that precipitate septic shock, acute-on-chronic liver failure (ACLF), organ dysfunction, and death [14,15]. They are associated with a four-fold increase in mortality, with ~30% deaths at one month and an additional 30% within one year [4]. The CANONIC study identified infections as the leading trigger of ACLF, accounting for 30% of cases [15].

Delay in effective antibiotics increases mortality by up to 7.6% per hour within the first six hours, making appropriate empirical coverage the strongest predictor of short-term survival [16]. However, rising MDR organism prevalence, coupled with geographic and institutional variations in resistance patterns, renders standardised antibiotic protocols increasingly untenable [17]. Recent studies have identified key MDR organism risk factors: prior hospitalisation, norfloxacin prophylaxis, previous broad-spectrum antibiotic exposure, and healthcare-associated infection acquisition [12,18].

Despite recognition of this crisis, prospective data from the Indian subcontinent - a recognised AMR hotspot - remain limited. Existing studies have largely focused on specific infection types or employed retrospective designs. No prospective study from North India has comprehensively evaluated the spectrum of bacterial infections and associated resistance patterns in decompensated cirrhosis.

The present study was designed to comprehensively evaluate the clinical profile of hospitalised patients with decompensated cirrhosis, assess the prevalence and clinical consequences of bacterial infections - including in-hospital mortality, ICU admission, organ support requirements, and healthcare resource utilisation - and analyse the microbiological spectrum along with antimicrobial susceptibility patterns from blood, urine, and ascitic fluid.

## Materials and methods

Study design and setting

This single-centre prospective observational cohort study was conducted at the Department of Medical Gastroenterology, Artemis Health Institute, Gurugram, India - a tertiary care referral centre in North India. The study was approved by the Institutional Ethics Committee and conducted in accordance with the Declaration of Helsinki. Written informed consent was obtained from all participants.

Study population and duration

Consecutive patients admitted with decompensated cirrhosis were prospectively enrolled over 18 months (September 2022 to February 2024).

Inclusion criteria

Patients aged ≥18 years admitted with decompensated cirrhosis were included. Cirrhosis was diagnosed based on clinical features (palmar erythema, spider telangiectasias), laboratory evidence (thrombocytopenia, hypoalbuminaemia, prolonged prothrombin time/international normalised ratio (PT/INR)), and imaging findings (nodular liver, splenomegaly, collaterals, ascites). Decompensation was defined per Baveno VII criteria: overt ascites (serum ascites albumin gradient (SAAG) >1.1 g/dL), overt hepatic encephalopathy (West Haven grade ≥II), or variceal bleeding [7].

Exclusion criteria

The exclusion criteria were unwillingness to consent, HIV/AIDS, immunosuppressant use, or prior liver transplantation.

Sample size

The sample size was calculated to estimate proportions with acceptable precision. Assuming a 50% proportion (maximising variability), 7.5% absolute precision, and a 95% confidence level, the minimum required sample size was: $n = \frac{Z_{\alpha/2}^2 \, p \, (1 - p)}{d^2}$, where Zα/2 = 1.96 (for 95% confidence), p = 0.50 (expected proportion), and d = 0.075 (absolute precision). This yielded n = 171. To account for potential incomplete data, 176 consecutively admitted patients were enrolled (3% oversampling). All enrolled patients had complete data for primary outcomes, as these were captured during index hospitalisation; there were no withdrawals or losses to follow-up.

Data collection and clinical assessments

Demographic data (age and sex), anthropometric measurements, and detailed medical history, including aetiology of cirrhosis, comorbidities (diabetes mellitus, hypertension, coronary artery disease, chronic kidney disease, and hypothyroidism), and prior antibiotic exposure, were recorded at enrolment. Cirrhosis aetiology was classified as alcohol-related, hepatitis B virus (HBV), hepatitis C virus (HCV), autoimmune, non-alcoholic steatohepatitis (NASH), Wilson’s disease, or cryptogenic based on standard criteria. Clinical examination findings, including jaundice, ascites, hepatic encephalopathy (West Haven grade), variceal bleeding, and coagulopathy, were documented. Disease severity was assessed using Child-Turcotte-Pugh (CTP) and Model for End-Stage Liver Disease-sodium (MELD-Na) scores [2,7].

Laboratory investigations

Blood samples collected at admission were analysed for complete blood count, liver function tests (total bilirubin, direct bilirubin, aspartate aminotransferase (AST), alanine aminotransferase (ALT), alkaline phosphatase (ALP), gamma-glutamyl transferase (GGT), total protein, albumin), renal function tests (blood urea nitrogen and serum creatinine), serum electrolytes (sodium, potassium), coagulation profile (PT/INR), serum ammonia, and procalcitonin using standard automated methods.

Microbiological evaluation

Ascitic Fluid

Diagnostic paracentesis was performed in all patients with new-onset or worsening ascites, GI bleeding, shock, hepatic encephalopathy, or deteriorating organ function. Ascitic fluid (10-20 mL) was collected aseptically and inoculated onto blood agar, MacConkey agar, chocolate agar, and brain-heart infusion (BHI) broth. Incubation was at 37 ± 2°C for 16-18 hours (with CO₂ for chocolate agar). Subculture from broth was performed if no growth occurred. Spontaneous bacterial peritonitis (SBP) was diagnosed with positive culture and ascitic fluid polymorphonuclear neutrophils (PMN) count >250 cells/mm³ [19].

Blood Cultures

Blood (8-10 mL per bottle) was collected aseptically and inoculated into BD BACTEC aerobic and anaerobic bottles, incubated in the BD BACTEC FX automated system (Becton, Dickinson and Company (BD), Franklin Lakes, NJ, USA) for up to five days. Positive samples were subcultured on blood agar and MacConkey agar. Identification and susceptibility testing were performed using VITEK-2 (bioMérieux, Marcy-l’Étoile, France) per CLSI guidelines.

Urine Cultures

Clean-catch midstream urine (10 mL) was inoculated on cystine-lactose-electrolyte-deficient (CLED) agar using a calibrated loop (0.001 mL) and incubated at 37 ± 2°C for 16-18 hours. Significant bacteriuria was defined as ≥10⁵ CFU/mL [20].

Other Infections

Pneumonia, skin and soft tissue infections (SSTIs), and others were diagnosed using conventional clinical, radiological, and microbiological criteria. Respiratory tract infection (RTI) was diagnosed based on symptoms, chest radiograph findings, and sputum culture when available. SSTI was diagnosed clinically.

Definitions

Systemic inflammatory response syndrome (SIRS) was defined as ≥2 of the following: heart rate >90/min, respiratory rate >20/min or PaCO₂ <32 mmHg, WBC >12,000/mm³ or <4,000/mm³ or >10% bands, temperature >38°C or <36°C.

MDR organisms were defined as non-susceptibility to ≥1 agent in ≥3 antimicrobial categories, including β-lactams [6]. Extensively drug-resistant (XDR) organisms were defined as non-susceptibility to ≥1 agent in all but ≤2 categories [21].

Hepatorenal syndrome-acute kidney injury (HRS-AKI) was defined per International Club of Ascites criteria [22].

The primary outcomes were infection prevalence, infection types, microbiological profile, and antimicrobial susceptibility. The secondary outcomes were in-hospital mortality, ICU admission, dialysis, mechanical ventilation, and AKI/HRS.

Statistical analysis

Data were analysed using IBM SPSS Statistics for Windows, Version 26 (Released 2018; IBM Corp., Armonk, NY, USA). Normality was assessed using the Kolmogorov-Smirnov test. Parametric continuous data (mean ± SD) were compared using Student’s t-test; non-parametric data (median and IQR) using the Mann-Whitney U test. Categorical data (numbers and percentages) were compared using chi-square or Fisher’s exact test. Variables with p < 0.05 in univariate analysis were included in multivariate logistic regression (adjusted for age, sex, and MELD-Na score) to identify independent predictors of infection and mortality. Odds ratios with 95% CI were reported. Two-tailed p < 0.05 was considered significant.

Ethical considerations

Artemis Health Sciences-Institutional Ethics Committee approval was obtained (approval number: 2023; dated 01/02/2023). Written informed consent was obtained from all participants. The study adhered to the Declaration of Helsinki and ICMR ethical guidelines.

## Results

Patient characteristics and infection prevalence

A total of 176 hospitalised patients with decompensated cirrhosis were enrolled in this study. The cohort comprised 122 men (69.3%) and 54 women (30.7%). Bacterial infections were documented in 82 patients (46.6%), while the remaining 94 patients (53.4%) had no clinical or microbiological evidence of infection.

Infected patients were significantly younger than their non-infected counterparts, with mean ages of 48.5 ± 13.0 years and 53.8 ± 12.3 years, respectively (p = 0.003). Male sex was more prevalent in the infected group, accounting for 63 infected patients (76.8%), compared with 59 non-infected patients (62.8%; p = 0.044).

Regarding cirrhosis aetiology, alcohol-related liver disease predominated in both groups. It was responsible for cirrhosis in 51 infected patients (62.2%) and 38 non-infected patients (40.4%). NASH was the second most common cause, affecting 21 infected patients (25.6%) and 34 non-infected patients (36.2%). Other aetiologies included hepatitis B, hepatitis C, autoimmune hepatitis, Wilson’s disease, and cryptogenic cirrhosis, each constituting less than 5% of the cohort.

As shown in Table 1, infected patients demonstrated a significantly higher prevalence of nearly all cirrhosis-related complications. Hepatic encephalopathy was present in 63 infected patients (76.8%) but in only 23 non-infected patients (24.5%; p < 0.001). Similarly, HRS-AKI occurred in 50 infected patients (61.0%) compared with 29 non-infected patients (30.9%; p < 0.001). Variceal bleeding was documented in 44 infected patients (53.7%) versus 28 non-infected patients (29.8%; p = 0.001), and ascites was present in 73 infected patients (89.0%) versus 71 non-infected patients (75.5%; p = 0.021). Jaundice showed a trend toward higher frequency in the infected group (81.7% vs. 69.1%), but this difference did not reach statistical significance (p = 0.056).

**Table 1:** Baseline Characteristics and Clinical Complications Data are presented as mean ± standard deviation (SD) for age and as number (percentage) for categorical variables. Between-group comparisons were performed using an independent t-test (for age) and the chi-square test (for all categorical variables). Statistical significance was set at p < 0.05. HRS-AKI: hepatorenal syndrome-acute kidney injury

Parameter	With Infection (n = 82)	Without Infection (n = 94)	Test Statistic	p-value
Age (years), mean ± SD	48.46 ± 12.96	53.80 ± 12.34	t = 2.98	0.003
Male sex, n (%)	63 (76.8%)	59 (62.8%)	χ² = 4.06	0.044
Cirrhosis complications, n (%)				
- Ascites	73 (89.0%)	71 (75.5%)	χ² = 5.36	0.021
- Variceal bleeding	44 (53.7%)	28 (29.8%)	χ² = 10.54	0.001
- Hepatic encephalopathy	63 (76.8%)	23 (24.5%)	χ² = 49.28	<0.001
- HRS-AKI	50 (61.0%)	29 (30.9%)	χ² = 16.51	<0.001
- Jaundice	67 (81.7%)	65 (69.1%)	χ² = 3.65	0.056

Infected patients demonstrated a significantly higher prevalence of all cirrhosis-related complications except jaundice. Hepatic encephalopathy was present in 63 (76.8%) infected versus 23 (24.5%) non-infected patients (χ² = 49.28, p < 0.001). Similarly, HRS-AKI occurred in 50 (61.0%) infected patients compared with 29 (30.9%) non-infected patients (χ² = 16.51, p < 0.001). Variceal bleeding (χ² = 10.54, p = 0.001) and ascites (χ² = 5.36, p = 0.021) were also significantly more frequent in the infected group.

Laboratory parameters and disease severity

Table 2 summarises the haematological, biochemical, and severity score comparisons between the two groups. Haematological evaluation revealed that infected patients had significantly higher total leukocyte counts, with a mean of 16.58 × 10³/mm³ compared with 7.78 × 10³/mm³ in non-infected patients (p < 0.001). Platelet counts were lower in the infected group (mean, 0.85 vs. 0.97 lacs/mm³; p = 0.017). Haemoglobin levels, however, were comparable between the two groups (p = 0.754).

**Table 2:** Laboratory Parameters and Severity Scores Data are presented as mean ± standard deviation (SD) for continuous variables and as number (percentage) for categorical variables. Between-group comparisons were performed using an independent t-test (for continuous variables) and the chi-square test (for categorical variables). Statistical significance was set at p < 0.05. TLC: total leukocyte count; SGPT: serum glutamate pyruvate transaminase; INR: international normalised ratio; CTP: Child-Turcotte-Pugh; MELD-Na: Model for End-Stage Liver Disease-sodium

Parameter	With Infection (n = 82)	Without Infection (n = 94)	Test Statistic	p-value
Haematological, mean ± SD				
Haemoglobin (g/dL)	9.02 ± 1.74	8.96 ± 1.93	t = 0.31	0.754
TLC (×10³/mm³)	16.58 ± 19.77	7.78 ± 4.53	t = 4.24	<0.001
Platelets (lacs/mm³)	0.85 ± 0.36	0.97 ± 0.38	t = 2.41	0.017
Biochemical, mean ± SD				
Total bilirubin (mg/dL)	7.11 ± 6.92	5.83 ± 7.20	t = 2.78	0.006
SGPT (U/L)	61.01 ± 51.74	43.14 ± 29.24	t = 3.19	0.002
Serum creatinine (mg/dL)	2.31 ± 1.67	1.32 ± 1.09	t = 5.62	<0.001
Serum albumin (g/dL)	2.65 ± 0.48	2.83 ± 0.38	t = 3.44	0.001
Serum ammonia (μmol/L)	94.20 ± 54.56	58.70 ± 48.74	t = 4.86	<0.001
Procalcitonin (ng/mL)	6.14 ± 9.33	1.51 ± 6.72	t = 3.94	<0.001
INR	2.34 ± 1.04	1.79 ± 0.76	t = 4.40	<0.001
Severity scores, n (%)				
CTP class C (>10)	77 (93.9%)	53 (56.4%)	χ² = 32.54	<0.001
MELD-Na >21	65 (79.3%)	35 (37.2%)	χ² = 31.81	<0.001

Biochemical parameters showed marked differences. Infected patients had significantly higher mean total bilirubin levels (7.11 vs. 5.83 mg/dL; p = 0.006) and SGPT levels (61.01 vs. 43.14 U/L; p = 0.002). Serum creatinine, a marker of renal dysfunction, was substantially elevated in infected patients (mean, 2.31 vs. 1.32 mg/dL; p < 0.001). Conversely, serum albumin was lower in the infected group (mean, 2.65 vs. 2.83 g/dL; p = 0.001), reflecting poorer synthetic function.

Inflammatory and coagulation markers were also significantly deranged in infected patients. Serum ammonia levels were markedly higher (mean, 94.20 vs. 58.70 μmol/L; p < 0.001), as were procalcitonin levels (mean, 6.14 vs. 1.51 ng/mL; p < 0.001). The INR was prolonged in infected patients (mean, 2.34 vs. 1.79; p < 0.001).

Disease severity, as measured by both CTP and MELD-Na scores, was substantially greater among infected patients. Nearly all infected patients, 77 (93.9%), were classified as CTP class C, compared with only 53 non-infected patients (56.4%; p < 0.001). Similarly, 65 infected patients (79.3%) had MELD-Na scores exceeding 21, whereas only 35 non-infected patients (37.2%) fell into this high-risk category (p < 0.001).

Clinical outcomes and resource utilisation

Table 3 presents the clinical outcomes and resource utilisation data. Patients with bacterial infections required intensive care substantially more often than those without infection. ICU admission was necessary for 57 infected patients (69.5%) but for only 28 non-infected patients (29.8%; p < 0.001). Mechanical ventilation was required in 52 infected patients (63.4%) compared with just eight non-infected patients (8.5%; p < 0.001). Haemodialysis was initiated in 31 infected patients (37.8%) versus seven non-infected patients (7.4%; p < 0.001).

**Table 3:** Clinical Outcomes and Resource Utilisation Data are presented as number (percentage) of patients. Between-group comparisons were performed using the chi-square test. Statistical significance was set at p < 0.05. ICU: intensive care unit; SIRS: systemic inflammatory response syndrome

Parameter	With Infection (n = 82)	Without Infection (n = 94)	Test Statistic	p-value
ICU admission, n (%)	57 (69.5%)	28 (29.8%)	χ² = 28.54	<0.001
Mechanical ventilation, n (%)	52 (63.4%)	8 (8.5%)	χ² = 62.41	<0.001
Haemodialysis, n (%)	31 (37.8%)	7 (7.4%)	χ² = 25.17	<0.001
In-hospital mortality, n (%)	31 (37.8%)	3 (3.2%)	χ² = 35.29	<0.001
SIRS present, n (%)	63 (76.8%)	36 (38.3%)	χ² = 26.83	<0.001
Prior antibiotic exposure, n (%)	70 (85.4%)	35 (37.2%)	χ² = 42.61	<0.001
Follow-up admission, n (%)	64 (78.0%)	36 (38.3%)	χ² = 28.90	<0.001

In-hospital mortality was dramatically higher in the infected group. A total of 31 infected patients (37.8%) died during their hospital stay, compared with only three non-infected patients (3.2%; p < 0.001). SIRS was present in 63 infected patients (76.8%) but in only 36 non-infected patients (38.3%; p < 0.001). Prior antibiotic exposure was reported in 70 infected patients (85.4%) versus 35 non-infected patients (37.2%; p < 0.001). Finally, 64 infected patients (78.0%) required follow-up admission compared with 36 non-infected patients (38.3%; p < 0.001).

Infection types and microbiological profile

Among the 82 patients with documented bacterial infections, RTI was the most common, affecting 22 patients (26.8%). Urinary tract infection (UTI) followed closely, occurring in 21 patients (25.6%). SBP was diagnosed in 17 patients (20.7%), while sepsis without an identifiable primary source was present in 15 patients (18.3%). SSTI was the least frequent, affecting seven patients (8.5%).

Table 4 provides the distribution of these infection types alongside their culture positivity rates. Overall, culture positivity was observed in 40 of the 82 infected patients (48.8%). The highest culture positivity rate was seen in sepsis cases, where 10 of 15 patients (66.7%) had positive cultures, followed by SSTI (4 of 7 patients, 57.1%) and UTI (11 of 21 patients, 52.4%). RTI and SBP had lower culture positivity rates of 40.9% (9 of 22) and 35.3% (6 of 17), respectively. Culture positivity differed across infection types. Sepsis demonstrated a significantly higher culture positivity rate compared to other infection types (10/15, 66.7%; p = 0.013), whereas differences for other infection types were not statistically significant. However, these comparisons should be interpreted with caution due to small sample sizes in certain subgroups, particularly SSTI (n = 7) and SBP (n = 17).

**Table 4:** Distribution of Infections and Bacterial Isolates Data are presented as number (percentage) of patients with each infection type and number (percentage) with positive cultures. Between-group comparisons of culture positivity rates across infection types were performed using the chi-square test. Statistical significance was set at p < 0.05 (bold values). Predominant isolates are presented as number of isolates within each infection type (e.g., UTI isolates n = 11, RTI n = 9, SBP n = 6, sepsis n = 10, SSTI n = 4), and do not represent percentages of total isolates. RTI: respiratory tract infection; UTI: urinary tract infection; SBP: spontaneous bacterial peritonitis; SSTI: skin and soft tissue infection; CONS: coagulase-negative staphylococci; *E. coli*: *Escherichia coli*

Infection Type	Total, n (%)	Culture Positive, n (%)	χ²	p-value	Predominant Isolates (n)
RTI	22 (26.8%)	9 (40.9%)	2.34	0.126	*Klebsiella* (2), *Acinetobacter* (2), *Streptococcus* (2)
UTI	21 (25.6%)	11 (52.4%)	4.67	0.031	*E. coli* (7), *Klebsiella* (3), *Enterococcus* (1)
SBP	17 (20.7%)	6 (35.3%)	0.89	0.345	*E. coli* (2), *Klebsiella* (2), *Enterococcus* (1), *Proteus *(1)
Sepsis	15 (18.3%)	10 (66.7%)	6.23	0.013	*Enterococcus* (3), CONS (3), *E. coli* (1), *Pseudomonas* (2), *Acinetobacter* (1)
SSTI	7 (8.5%)	4 (57.1%)	2.01	0.156	*Pseudomonas* (2), *Staphylococcus* (2)
Total	82 (100%)	40 (48.8%)	-	-	

Microbiological profile of isolates

A total of 40 bacterial isolates were recovered from culture-positive patients. As detailed in Table 4 (lower portion), Gram-negative bacteria predominated, accounting for 31 isolates (77.5%). *E. coli* was the single most common isolate, identified in 11 patients (27.5% of all isolates). *Klebsiella* species followed, with seven isolates (17.5%). *Pseudomonas* species and *Enterococcus* species were each recovered from five patients (12.5% each). Coagulase-negative staphylococci (CONS) were isolated from four patients (10.0%), *Acinetobacter* species from three patients (7.5%), and *Staphylococcus* species, *Streptococcus* species, and *Proteus* species from five patients collectively (12.5%).

Site-specific distribution revealed important patterns. Among UTI isolates (n = 11), *E. coli* was the dominant pathogen, accounting for 7/11 (63.6%). In SBP, *E. coli* and *Klebsiella* were equally represented, with two isolates each (33.3% each). RTI isolates were more diverse, with *Klebsiella*, *Acinetobacter*, and *Streptococcus* each contributing two isolates (22.2% each). SSTI isolates were split between *Pseudomonas* (two isolates, 50.0%) and *Staphylococcus* (two isolates, 50.0%). In sepsis cases, *Enterococcus* and CONS each accounted for three isolates (30.0% each).

AMR patterns

Table 5 presents the AMR profiles of the 40 bacterial isolates. Overall, drug resistance was observed in 23 isolates (57.5%). Of these, 18 isolates (45.0% of all isolates) met the criteria for MDR organisms, and five isolates (12.5% of all isolates) were classified as XDR. The remaining 17 isolates (42.5%) were non-resistant.

**Table 5:** Antimicrobial Resistance Patterns by Organism Data are presented as the number (percentage) of total isolates. Multidrug-resistant (MDR) organisms were defined as non-susceptibility to at least one agent in three or more antimicrobial categories [9]. Extensively drug-resistant (XDR) organisms were defined as non-susceptibility to at least one agent in all but two or fewer antimicrobial categories [9]. Between-group comparisons of resistance patterns across organisms were performed using the chi-square test. Statistical significance was set at p < 0.05. *Others include *Staphylococcus* spp., *Streptococcus* spp., and *Proteus* spp. CONS: coagulase-negative staphylococci; *E. coli*: *Escherichia coli*

Organism	Total Isolates, n (%)	MDR, n (%)	XDR, n (%)	Non-resistant, n (%)
E. coli	11 (27.5%)	6 (54.5%)	1 (9.1%)	4 (36.4%)
*Klebsiella* spp.	7 (17.5%)	3 (42.9%)	2 (28.6%)	2 (28.6%)
*Enterococcus* spp.	5 (12.5%)	3 (60.0%)	0 (0%)	2 (40.0%)
*Pseudomonas* spp.	5 (12.5%)	5 (100%)	0 (0%)	0 (0%)
*Acinetobacter* spp.	3 (7.5%)	1 (33.3%)	2 (66.7%)	0 (0%)
CONS	4 (10.0%)	0 (0%)	0 (0%)	4 (100%)
Others*	5 (12.5%)	0 (0%)	0 (0%)	5 (100%)
Total	40 (100%)	18 (45.0%)	5 (12.5%)	17 (42.5%)

Among *E. coli* isolates (n = 11), six (54.5%) were MDR and one (9.1%) was XDR. *Klebsiella* species (n = 7) showed MDR in three isolates (42.9%) and XDR in two isolates (28.6%). Alarmingly, all five *Pseudomonas* isolates (100%) were MDR. *Acinetobacter* species (n = 3) demonstrated MDR in one isolate (33.3%) and XDR in two isolates (66.7%). *Enterococcus* species (n = 5) were MDR in three isolates (60.0%). In contrast, all CONS, *Staphylococcus*, *Streptococcus*, and *Proteus* isolates were uniformly non-resistant.

Table 6 shows resistance patterns stratified by infection site. The highest MDR prevalence was observed in UTIs, where 8 of 11 isolates (72.7%) demonstrated MDR, followed by SSTI (2 of 4 isolates, 50.0%), sepsis (4 of 10 isolates, 40.0%), RTI (3 of 9 isolates, 33.3%), and SBP (1 of 6 isolates, 16.7%). XDR organisms were most frequent in SBP, affecting two of six isolates (33.3%), followed by RTI (1 of 9 isolates, 11.1%), sepsis (1 of 10 isolates, 10.0%), and UTI (1 of 11 isolates, 9.1%). No XDR organisms were identified in SSTI isolates. The differences in resistance patterns across infection sites were statistically significant for UTI compared with other sites (p = 0.009).

**Table 6:** Resistance Patterns by Infection Site Data are presented as the number (percentage) of isolates from each infection site. Multidrug-resistant (MDR) and extensively drug-resistant (XDR) organisms were defined per standard criteria [9]. Between-group comparisons of resistance patterns across infection sites were performed using the chi-square test. Statistical significance was set at p < 0.05 (bold values). UTI: urinary tract infection; SBP: spontaneous bacterial peritonitis; RTI: respiratory tract infection; SSTI: skin and soft tissue infection

Infection Site	Total Isolates, (n)	MDR, n (%)	XDR, n (%)	Non-resistant, n (%)	χ²	p-value
UTI	11	8 (72.7%)	1 (9.1%)	2 (18.2%)	9.45	0.009
SBP	6	1 (16.7%)	2 (33.3%)	3 (50.0%)	4.32	0.115
RTI	9	3 (33.3%)	1 (11.1%)	5 (55.6%)	3.21	0.201
SSTI	4	2 (50.0%)	0 (0%)	2 (50.0%)	2.89	0.236
Sepsis	10	4 (40.0%)	1 (10.0%)	5 (50.0%)	3.67	0.159
Total	40	18 (45.0%)	5 (12.5%)	17 (42.5%)	-	-

Site-specific resistance analysis revealed the highest MDR prevalence in UTI, with 8 (72.7%) of 11 isolates demonstrating MDR, followed by SSTI with two (50.0%), sepsis with four (40.0%), RTI with three (33.3%), and SBP with one (16.7%). XDR organisms were most frequent in SBP, affecting two (33.3%) isolates, followed by RTI with one (11.1%), sepsis with one (10.0%), and UTI with one (9.1%). No XDR organisms were identified in SSTI.

Key antibiotic susceptibility patterns

Table 7 provides detailed susceptibility data for the most common Gram-negative isolates against key antibiotics. For *E. coli* (n = 11), susceptibility was highest to colistin (11 isolates, 100%), meropenem (10 isolates, 90.9%), imipenem (9 isolates, 81.8%), piperacillin-tazobactam (8 isolates, 72.7%), and amikacin (7 isolates, 63.6%). In contrast, susceptibility to third-generation cephalosporins was poor, with only three isolates (27.3%) susceptible to ceftriaxone. Fluoroquinolone susceptibility was also low, with only five isolates (45.5%) susceptible to ciprofloxacin.

**Table 7:** Susceptibility of Common Gram-Negative Isolates to Key Antibiotics Data are presented as number (percentage) of isolates susceptible to each antibiotic. Susceptibility testing was performed using the VITEK-2 automated system (bioMérieux, Marcy-l’Étoile, France), following Clinical and Laboratory Standards Institute (CLSI) guidelines. Ceftriaxone was tested in only one of five Pseudomonas isolates (20%); the result shown (0%) reflects susceptibility in that single isolate. Susceptibility data should be interpreted cautiously due to the limited number of isolates for some organisms. *E. coli*: *Escherichia coli*

Antibiotic	*E. coli* (n = 11), n (%)	*Klebsiella* (n = 7), n (%)	*Pseudomonas* (n = 5), n (%)	*Acinetobacter* (n = 3), n (%)
Colistin	11 (100%)	6 (85.7%)	0 (0%)	3 (100%)
Meropenem	10 (90.9%)	4 (57.1%)	3 (60.0%)	0 (0%)
Imipenem	9 (81.8%)	5 (71.4%)	3 (60.0%)	1 (33.3%)
Amikacin	7 (63.6%)	5 (71.4%)	3 (60.0%)	3 (100%)
Piperacillin-tazobactam	8 (72.7%)	2 (28.6%)	3 (60.0%)	1 (33.3%)
Ceftriaxone	3 (27.3%)	2 (28.6%)	0 (0%)	0 (0%)
Ciprofloxacin	5 (45.5%)	2 (28.6%)	0 (0%)	0 (0%)

For *Klebsiella* species (n = 7), high susceptibility was observed to colistin (6 isolates, 85.7%), amikacin (5 isolates, 71.4%), and imipenem (5 isolates, 71.4%). Meropenem susceptibility was moderate, affecting four isolates (57.1%). Ceftriaxone and ciprofloxacin susceptibilities were poor, at 28.6% (2 isolates each).

*Pseudomonas* isolates (n = 5) showed susceptibility to polymyxins in four isolates (80.0%), with moderate susceptibility (60.0%) to carbapenems (meropenem and imipenem), aminoglycosides (amikacin), and piperacillin-tazobactam. Ceftriaxone is not typically used for *Pseudomonas* and showed no susceptibility in the single isolate tested.

*Acinetobacter* species (n = 3) demonstrated complete susceptibility (100%) to amikacin and colistin but poor susceptibility to other agents, including meropenem (0% susceptibility), imipenem (1 isolate, 33.3%), and piperacillin-tazobactam (1 isolate, 33.3%).

Non-MDR Gram-positive isolates (CONS, *Staphylococcus*, *Streptococcus*) demonstrated high susceptibility (exceeding 90%) to glycopeptides and oxazolidinones. However, 60% of *Enterococcus* isolates were MDR, warranting continued vigilance.

Predictors of infection and mortality

Multivariate logistic regression analysis was performed to identify independent predictors of bacterial infection among all 176 patients and predictors of in-hospital mortality among the 82 infected patients. The results are presented in Table 8.

**Table 8:** Independent Predictors of Infection and Mortality (Multivariate Logistic Regression) Multivariate logistic regression analysis was performed to identify independent predictors of bacterial infection (among all 176 patients) and in-hospital mortality (among the 82 infected patients). Variables with p < 0.05 in univariate analysis were included in the multivariate models, adjusted for age, sex, and MELD-Na score. Results are presented as adjusted odds ratios (ORs) with 95% confidence intervals (CIs) and Wald chi-square (χ²) statistics. Statistical significance was set at p < 0.05. INR: international normalised ratio; MELD-Na: Model for End-Stage Liver Disease-sodium

Outcome	Variable	Adjusted OR	95% CI	Wald χ²	p-value
Infection	INR	2.053	1.350-3.121	10.82	0.001
	Serum albumin	0.442	0.210-0.932	4.58	0.032
In-hospital mortality	Total bilirubin	1.140	1.045-1.245	8.96	0.003

After adjusting for age, sex, and MELD-Na score, two variables emerged as independent predictors of bacterial infection. Elevated INR was associated with a significantly increased risk of infection, with an adjusted odds ratio of 2.053 (95% confidence interval: 1.350 to 3.121; p = 0.001). Conversely, higher serum albumin was protective against infection, with an adjusted odds ratio of 0.442 (95% confidence interval: 0.210 to 0.932; p = 0.032), meaning that each 1 g/dL increase in albumin reduced the odds of infection by approximately 56%. Other variables, including serum sodium and total bilirubin, did not retain statistical significance as independent predictors after adjustment.

Among the 82 patients diagnosed with bacterial infections based on predefined clinical and microbiological criteria, elevated total bilirubin was the sole independent predictor of in-hospital mortality. Each 1 mg/dL increase in total bilirubin conferred a 14% increase in mortality risk, with an adjusted odds ratio of 1.140 (95% confidence interval: 1.045 to 1.245; p = 0.003).

## Discussion

This prospective observational study of 176 hospitalised patients with decompensated cirrhosis revealed several important findings. Bacterial infections, diagnosed using predefined clinical and microbiological criteria, were identified in 82/176 patients (46.6%). Culture positivity was observed in 40/82 infected patients (48.8%), consistent with the known frequency of culture-negative infections in cirrhosis. The overall infection prevalence remained within the 25%-47% range reported in the literature [23].

Patient characteristics and risk factors

Infected patients were significantly younger than non-infected patients. This finding aligns with recent data from the Indian subcontinent, which reported mean ages of approximately 47-48 years in infected ACLF cohorts [24,25]. Male predominance in the infected group is consistent with global trends, with previous large cohorts reporting similar distributions [26]. This sex distribution likely reflects higher rates of alcohol use among men, as well as potential differences in healthcare-seeking behaviour.

Infection was strongly associated with cirrhosis complications. Hepatic encephalopathy, HRS, and variceal bleeding were significantly more frequent among infected patients compared with non-infected patients. Prior studies have shown that infections exacerbate cognitive dysfunction in cirrhosis, a finding that may reverse with infection resolution [27]. The relationship between infection and variceal bleeding appears bidirectional: bleeding promotes bacterial translocation, while infections impair haemostasis and increase portal pressure [28]. Current European Association for the Study of the Liver (EASL) guidelines appropriately recommend antibiotic prophylaxis for all patients following variceal bleeding [29].

The high prevalence of HRS among infected patients appears greater than that reported in earlier studies [30]. Cirrhosis-associated circulatory dysfunction predisposes patients to infection-induced renal failure [31]. This finding underscores the need to actively evaluate for infection in any decompensated patient presenting with renal dysfunction.

Alcohol-related liver disease was the predominant aetiology in both groups, with a higher proportion observed among infected patients. These figures are consistent with prior Indian studies [32,33]. The higher prevalence of alcohol-related cirrhosis among infected patients may reflect alcohol-related immune dysfunction, which further compromises host defences in cirrhosis [34].

Laboratory parameters and disease severity

Infected patients exhibited significantly higher total leukocyte counts, a finding consistent with prior reports [35], which reflects systemic inflammation. Procalcitonin levels were markedly elevated in infected patients, supporting its role as a useful biomarker for bacterial infection in cirrhosis [36]. Disease severity was substantially greater in infected patients, with a higher proportion classified as CTP class C and having elevated MELD-Na scores compared with non-infected patients. These findings are consistent with previous studies that identified higher MELD scores as independent risk factors for infection [26] and underscore that advanced cirrhosis represents a high-risk state for infection-related complications, including ACLF [37].

Clinical outcomes

This finding is consistent with prior studies demonstrating substantially increased mortality associated with infection in cirrhosis [25,26,38].

Infection types and microbiological profile

RTIs were the most common infection type (26.8%), a finding that contrasts with studies from other regions where UTI or SBP predominated [27,38,39]. However, recent data suggest that pneumonia may be more frequent in Asian settings, whereas UTIs predominate in Europe and the Americas [25]. The observed SBP prevalence (20.7%) is consistent with global estimates ranging from 10% to 30% [40].

The overall culture positivity rate falls within the reported range, underscoring the diagnostic challenges unique to cirrhosis, where prior antibiotic exposure and ascitic fluid dilution may limit microbiological yield [26,41]. Gram-negative bacteria predominated (77.5% of isolates), with *E. coli *(27.5%) and *Klebsiella* species (17.5%) as the most common isolates. This pattern is consistent with prior global and regional data [26]. The presence of *Enterococcus* species (12.5%) among common isolates reflects the evolving epidemiology toward Gram-positive organisms, a shift likely driven by increased invasive procedures and antibiotic exposure [38,42].

AMR pattern

The high overall drug resistance rate (57.5% of isolates), comprising 45.0% MDR and 12.5% XDR organisms, represents a major clinical concern. These figures are consistent with data from the Indian subcontinent reporting high rates of MDR infections and carbapenem resistance in cirrhosis [32,43]. However, organism-specific susceptibility patterns should be interpreted cautiously due to the relatively small number of isolates available for certain pathogens. Global data identify this region as having one of the highest MDR and XDR burdens [26].

Site-specific resistance patterns carry direct therapeutic implications. MDR prevalence appeared highest in UTIs (8/11, 72.7%), followed by SSTI (2/4, 50.0%), sepsis (4/10, 40.0%), RTI (3/9, 33.3%), and SBP (1/6, 16.7%), although interpretation is limited by small subgroup sizes. In contrast, XDR organisms were most frequent in SBP (33.3%, 2 of 6 isolates). These findings suggest limited utility of cephalosporins and fluoroquinolones for empirical treatment of suspected UTI in this setting.

Resistance among Gram-negative isolates was substantial. *E. coli* and *Klebsiella* species showed poor susceptibility to ceftriaxone (27.3% and 28.6%, respectively) and to fluoroquinolones (45.5% and 28.6%, respectively), limiting the utility of traditional first-line options. In contrast, carbapenems, aminoglycosides, and colistin demonstrated relatively preserved activity against most tested isolates. These patterns are consistent with reports of reduced efficacy of cephalosporin-based regimens in high-resistance settings [43]. Gram-positive organisms remained uniformly susceptible to glycopeptides and oxazolidinones, although a notable proportion of *Enterococcus* isolates were MDR (60.0%), warranting continued vigilance.

Predictors of infection and mortality

Multivariate analysis identified elevated INR (adjusted OR 2.053; p = 0.001) and hypoalbuminemia (adjusted OR 0.442; p = 0.032) as independent predictors of bacterial infection. Both parameters reflect impaired hepatic synthetic function. Prior studies have similarly shown that markers of advanced liver dysfunction, including components of the CTP and MELD scores, are associated with increased infection risk [26,38].

Among infected patients, elevated total bilirubin independently predicted mortality (adjusted OR 1.140; p = 0.003). This finding is consistent with previous reports that incorporated bilirubin into prognostic models [27,44] and likely reflects infection-related cholestasis and worsening hepatic dysfunction, both of which are associated with poor outcomes.

Clinical implications

These findings carry immediate clinical relevance. First, the high MDR and XDR prevalence mandates reevaluation of empirical antibiotic protocols. Third-generation cephalosporins and fluoroquinolones, currently recommended for community-acquired infections [24,30], appear inadequate in this setting. Piperacillin-tazobactam, carbapenems, or amikacin may be more appropriate empirical choices while awaiting culture results, particularly for healthcare-associated infections. Second, infection prevention strategies, including minimising unnecessary admissions, reducing invasive procedures, strengthening antimicrobial stewardship, and implementing strict infection control measures, are urgently needed [25,32]. Effective antimicrobial stewardship programmes, supported by structured, data-driven quality improvement approaches, may further facilitate appropriate empirical antibiotic selection and help limit the emergence and spread of AMR [44]. Third, simple bedside markers (INR, albumin, bilirubin) can identify patients at the highest infection and mortality risk, enabling targeted prevention and intensive monitoring.

Limitations

Several limitations warrant acknowledgement. This single-centre study may limit generalisability to other populations or healthcare settings. The modest sample size (n = 176) and limited number of culture-positive isolates for certain organisms precluded robust subgroup analyses by infection type or pathogen. Culture negativity in 51.2% of infected patients, partly attributable to prior antibiotic exposure, may have introduced selection bias in resistance analysis [26]. The study lacked data on proton pump inhibitor use, nutritional status, and timing of antibiotic initiation - factors that may influence outcomes. Finally, SIRS criteria may be confounded by cirrhosis itself, as decompensated patients can exhibit SIRS without infection [31].

Future directions

Multicentre prospective studies with larger cohorts are needed to validate these findings and establish regional antibiograms to guide empirical therapy. Longitudinal studies examining the impact of antimicrobial stewardship and structured quality improvement initiatives on resistance patterns and clinical outcomes are also essential. Research into novel biomarkers for early infection detection in culture-negative patients, as well as preventive strategies including vaccination and gut microbiome modulation, may help reduce infection burden. Given the escalating MDR crisis, particularly in India, urgent public health interventions addressing antibiotic overuse in both community and healthcare settings are imperative.

## Conclusions

This study demonstrates that bacterial infections are highly prevalent among hospitalised patients with decompensated cirrhosis and are associated with significantly worse clinical outcomes. The high burden of AMR substantially limits the effectiveness of conventional empirical therapies, highlighting the urgent need for region-specific treatment strategies.

Simple clinical and biochemical markers may aid in early risk stratification and timely intervention. Strengthening antimicrobial stewardship and implementing effective infection prevention strategies are essential to improve outcomes in this vulnerable population.
